# Prevalence of Self-medication for Acid Peptic Disease amongst People of Manawa, Lahore

**DOI:** 10.7759/cureus.6817

**Published:** 2020-01-30

**Authors:** Irsa R Chattha, Sehrish Zaffar, Shoaib Tariq, Waqar Ahmed Siddiqui, Kamran Zaman, Rizwana Kamran, Aisha Talat, Hira Tanveer

**Affiliations:** 1 Pharmacology, CMH Lahore Medical College (NUMS), Lahore, PAK; 2 Internal Medicine, Indus Hospital Manawan, Lahore, PAK; 3 Medical Education and Simulation, CMH Lahore Medical and Dental College, National University of Medical Sciences, Lahore, PAK

**Keywords:** acid peptic disease, self medication

## Abstract

Introduction

Acid peptic disease is a common disorder, affecting millions of people worldwide. Its pharmacological management includes proton pump inhibitors, H_2_ blockers, antacids and mucosal protective agents. Multiple studies in Pakistan have shown increased tendency of population for self-medication. This has serious implications regarding false diagnoses, misuse of drugs and occurrence of adverse effects. The objective of this study was to estimate the prevalence of self-medication among the people of Manawa, Lahore.

Methods

A cross-sectional study was conducted between January 2019 and June 2019 amongst the general population of Manawa, Lahore. Patients with the typical presentation of acid peptic disease were included in the study. A total of 500 people, fulfilling the inclusion criteria, were chosen. A questionnaire form was adapted from similar studies performed on self-medication. It was divided into two parts. First part included the social and demographic data while the second part consisted of details about the prevalence and factors related to the practice of self-medication amongst acid peptic disease (APD) patients. The data collected was transcribed into SPSS 22.0 (IBM Corp., Armonk, NY) for statistical analysis. All the categorical variables were analyzed as frequencies and percentages. No statistical comparisons were done as it was a descriptive, exploratory study.

Results

Out of 500 participants, 404 were females and 96 were males. Most of them were middle-aged (53%) and illiterate (68.4%). Number of participants who practiced self-medication was found to be 313 (62.6%). The highest prevalence of self-medication was found for proton pump inhibitors (43.1%), followed by antacids (23.6%), unknown homeopathic and Hakeem medicine (20.4%) and H_2_ blockers (12.8%). The most common reasons stated for self-medication included inability to afford medical consultation (44.2%), lack of knowledge about drug side effects (35.8%), easy access to Hakeem and homeopathic medicines (27.6% and 4.8%, respectively) and inaccessibility to doctors (19.2%).

Conclusion

A large majority of people in Manawa practise self-medication for acid peptic disease, owing to multiple reasons.

## Introduction

Acid peptic disease (APD) comprises a group of disorders of the gastrointestinal tract that include gastroesophageal reflux disease (GERD), peptic ulcer disease (PUD) and dyspepsia. It occurs due to weakening of the mucosal defenses or increased secretion of acid or pepsin from either gastric or duodenal mucosa [1]. Acid peptic disease is a common disorder, affecting millions of people worldwide and is an important cause of both mortality and morbidity. Its prevalence is about 1.5% in the population of USA in 1995, with more than half, being cases of GERD [2].

Patients with GERD normally complain of acid reflux, regurgitation, chest pain, cough and dysphagia. If the first two symptoms are present in the patient, the accuracy of clinical diagnosis is 90% [3]. Risk factors that lead to the development of APD include various foods, especially those that are high in fat content, beverages such as coffee and tea, smoking, alcohol and use of medication such as nonsteroidal anti-inflammatory drugs (NSAIDs) [4].

Diet and lifestyle modification, therefore, plays a pivotal role in the treatment of APD, in addition to the pharmacological therapy. Measures such as short interval meals, decreased intake of alcohol, smoking and spicy food, increased intake of fiber containing diet, dinner three hours before bedtime and raised head end while sleeping have proved useful in alleviation of APD symptoms [5].

Pharmaceutical treatment includes proton pump inhibitor, H_2_ blocker, antacids and mucosal protective agents. Proton pump inhibitors are the most commonly used drug and are more effective than H_2_ receptor blockers. They inhibit the H-K ATPase pump, leading to a decreased secretion of acid in gastric mucosa. It is normally given for 4-8 weeks and can be given for an extended period of time depending upon severity of the disease. Second class of drug that is commonly prescribed is H_2_ receptor blocker while antacids are used for temporary relief of symptoms [6].

Self-medication has become an increasingly common trend over the last few years. An inclination to self-diagnose the disease and thereby treating it as well, has become a regular practice amongst the masses. Generally, people are not aware of the adverse effects of the drugs or the recommended dosage. This can often lead to serious consequences [7]. For example, excessive use of antacids can lead to milk alkali syndrome characterized by a triad of metabolic alkalosis, hypercalcemia and renal insufficiency [8]. Consuming proton pump inhibitors over a prolonged period of time can cause osteoporosis, vitamin B12 deficiency, upper respiratory tract infections and increased incidence of clostridium difficile infection [9]. Similarly, H_2_ receptor blockers can prolong the half-life of other drugs due to enzyme inhibition and result in serious toxicity such as increased risk of major bleeding when given along with warfarin [10].

The purpose of this study was to estimate the prevalence of self-medication among the people of Manawa, Lahore as well as to observe the reasons leading to such practices.

## Materials and methods

A cross-sectional study was conducted between January 2019 and June 2019 amongst the general population of Manawa, Lahore. Patients with the typical presentation of acid peptic disease, according the PAGI-SYM, were included in the study [11]. A total of 500 people, fulfilling the inclusion criteria, were chosen. These included 96 males and 404 females, belonging to varying age groups. Patients who were already consulting a physician were excluded. Those suffering from any other disease, were also excluded.

A pretested, pre-designed questionnaire form was adapted from similar studies performed on self-medication [12-14]. It was divided into two parts. First part included the social and demographic data while the second part consisted of details about the prevalence and factors related to the practice of self-medication amongst APD patients.

Informed consent was taken from each participant before commencement of the study. The participants were asked to fill the questionnaire form, with the help of the administrator, wherever required. The study was carried out in accordance with the guidelines detailed in Good Epidemiology Practice, Declaration of Helsinki and local laws and regulations [15,16].

The data collected was transcribed into SPSS 22.0 (IBM Corp., Armonk, NY) for statistical analysis. All the categorical variables were analyzed as frequencies and percentages. No statistical comparisons were done as it was a descriptive, exploratory study.

## Results

Total number of participants were 500. Of these, 96 were males and 404 were females. Patients within the age group of 11-70 years were included in the study (Table 1).

**Table 1:** Frequency of participants belonging to different age groups

Age (years)	Number of participants (n = 500)	Percentage of participants (%)
11-30	97	19.4
31-50	265	53
51-70	127	25.4
70 and above	11	2.2

A vast majority of these patients were illiterate, while remaining patients had varying level of qualification (Table 2).

**Table 2:** Literacy status of participants

Level of qualification	Number of participants (n = 500)	Percentage of participants (%)
Illiterate	342	68.4
Primary education	51	10.2
Secondary education	50	10
Matric/Inter	45	9
Graduation	12	2.4

The majority of patients belonged to low socioeconomic strata. Table 3 represents the monthly income of the patients.

**Table 3:** Monthly income of participants

Monthly income (PKR)	Number of participants (n = 500)	Percentage of participants (%)
<5000	45	9
5000-15000	266	53.2
15000-25000	118	23.6
>25000	71	14.2

The percentage of self-medicating individuals was found to be 62.6% (313). Table 4 represents the drugs that are being used for self-medication.

**Table 4:** Drugs used for self-medication of acid peptic disease

Drugs	Number of participants (n = 313)	Percentage of participants (%)
Proton pump inhibitors	135	43.1
Antacids	74	23.6
H2 receptor blockers	40	12.8
Unknown drugs	64	20.4

The reasons cited for self-medication are tabulated as follows (Table 5).

**Table 5:** Reasons for self-medication

Reason for self-medication	Number of participants (n = 313)	Percentage of participants (%)
Affordability issue	139	44.2
Unawareness of side effects	112	35.8
Prefer Hakeem medication	86	27.6
Inaccessibility to doctors	60	19.2
Prefer Homeopathic medication	15	4.8

## Discussion

Self-medication has become a popular trend in recent years, locally as well as globally [17]. Multiple studies conducted in Pakistan have shown increased tendency of population towards self-diagnosing and treatment. This has serious implications regarding false diagnoses, misuse of drugs and occurrence of adverse effects [18].

The incidence of acid peptic disease has increased markedly over the last few years, mainly due to change in dietary habits and lifestyle. Unfortunately, gastric upset is not treated seriously until severe symptoms emerge, such as hematemesis or gastric perforation [19]. People generally tend to treat mild dyspepsia and heartburn with home remedies. Even worse, when the allopathic treatment becomes unavoidable, people hesitate visiting a doctor and instead, use over the counter available medicines upon recommendation of a friend or relative. Such practices have led to serious consequences including worsening of the disease or occurrence of life-threatening adverse effects due to wrong self-medication. Currently, there is a lack in studies regarding use of nonprescription medicines for peptic ulcer disease.

This study was conducted to observe the trend of self-medication for acid peptic disease in general population of Manawa, Lahore. According to this study, 62.6% of the participants opted for self-medication for the relief of acid peptic disease. This is comparable to the results of Singh et al., in which 61.5% undergraduate medical students were found to practise self-medication [20]. Similarly, prevalence of self-medication was found to be 59% in Pokhara Valley, Nepal [21]. In Pakistan, much higher prevalence of self-medication has been shown in studies conducted by Zafar et al. (76%) and Afridi et al. (84.8%) [22,23].

Amongst the different drugs available, proton pump inhibitors (PPIs) were the most consumed medication (43.1%), which is comparable to another study conducted in Pakistan, by Butt and Hashemy [24]. Various studies have demonstrated that long-term use of proton pump inhibitors can lead to vitamin B12 deficiency, development of osteoporosis and increased risk of upper respiratory tract infections and clostridium difficile infection [9]. Similarly, 23.6% individuals were found consuming antacids without prescription. This can lead to development of serious complications such as milk alkali syndrome and acute pancreatitis [8].

Most of the participants were females (80.8%) and belonged to the age group of 31-50 years (53%). It is an alarming concern that a vast majority of participants were illiterate (68.4%). Illiteracy is the root of lack of awareness amongst people and diverts the population towards falsely acclaimed Homeopathic and Hakeem medications. In this study, 35.8% of self-medicating participants admitted that they have no awareness about the potential complications of the drugs they are using, while Hakeem and homeopathic medicine was preferred by 27.6% and 4.8% individuals, respectively. Moreover, 85.8% of participants had a monthly income of less than 25,000 Pakistani rupees. This signifies the impact of poverty on health status of the people. In this study, 44.2% of individuals, practicing self-medication, stated that scarcity of funds was the reason behind not consulting a physician. Another reason for self-medication was found to be inaccessibility to the doctors (19.2%), resulting from a lack of provision of healthcare facilities within the range of each residential area.

This study had a limitation that only a small residential area of Lahore district, Manawa, was included because of easy access. Multiple areas could not be included due to scarcity of funds and resources.

## Conclusions

A strong trend of self-medication for acid peptic disease was observed amongst the people of Manawa. This was generally attributed to lack of awareness of drug complications, lack of funds to afford medical consultation and difficulty access to doctors. It is of utmost importance to increase the literacy rate in this region. Efforts need to be done at individual and state level to encourage education. Awareness about drug adverse effects should be spread through multiple means such as social media and free medical camps. Most importantly, self-medication should be discouraged by making strong legislations regarding the sale of drugs without prescription.
